# The Use of Intraoperative Cell Salvage in Two-Stage Revision of Septic Hip Arthroplasties: A Double-Center Retrospective Study

**DOI:** 10.3390/antibiotics12060982

**Published:** 2023-05-30

**Authors:** Lara Krüger, André Strahl, Leon-Gordian Koepke, Bernd Fink, Frank Timo Beil, Jan Hubert

**Affiliations:** 1 Department of Trauma and Orthopaedic Surgery, Division of Orthopaedics, University Medical Centre Hamburg-Eppendorf, 20246 Hamburg, Germany; 2 Department of Trauma and Orthopaedic Surgery, Division of Spine Surgery, University Medical Centre Hamburg-Eppendorf, 20246 Hamburg, Germany; 3 Department of Joint Replacement, General and Rheumatic Orthopaedics, Orthopaedic Clinic Markgröningen, 71706 Markgröningen, Germany

**Keywords:** periprosthetic joint infection, cell salvage, patient blood management, septic surgery, revision arthroplasty

## Abstract

(1) Background: intraoperative cell salvage (ICS) devices can provide a valuable contribution to patient blood management. An infection of the surgical site presents a formal contraindication to the use of ICS. To date, there is no recommendation for the use of ICS in the context of reimplantation in two-stage septic exchange arthroplasty. (2) Methods: at two hospitals of maximum endoprosthetic care, a retrospective evaluation of patients who had received ICS blood during reimplantation of hip arthroplasties was performed. Patients’ and surgical characteristics, intraoperative cultures, and the occurrence of septic complications in the short- and long-term follow-up were recorded. (3) Results: 144 patients were included. Detection of positive cultures during reimplantation occurred in 13 cases. A total of 127 patients showed no complication, 8 patients showed a non-specific septic complication, 6 patients a local persistence of infection, and 3 patients a possible bloodstream-associated infection. No significant correlation was found between the occurrence of complications and the detection of positive intraoperative cultures. (4) Conclusions: no clustering of septic complications due to the use of ICS during reimplantation was found. In the risk-benefit analysis, we considered the use of ICS during reimplantation to be indicated in terms of patient blood management, while the safety of the procedure during septic first-stage resection arthroplasty or septic one-stage exchange arthroplasty was not investigated. Given the paucity of comparative literature, further studies are needed on ideal patient blood management in the setting of septic revision arthroplasty.

## 1. Introduction

According to the definition of the World Health Organization (WHO), anemia is defined as a hemoglobin concentration (Hb value) of <13 g/dL (8.07 mmol/L) in men and <12 g/dL (7.45 mmol/L) in women [1]. The impact of anemia on the clinical outcome of surgical patients may be dramatic. Perioperatively untreated anemia has been shown to be an independent risk factor for increased morbidity and mortality as well as a prolonged hospital stay [2,3,4]. During and after revision arthroplasty and especially in septic revision arthroplasty, blood loss often occurs and allogeneic red blood cell transfusion (ARBCT) or intraoperative cell salvage (ICS) can be used to address this situation.

ARBCT is associated with a transfusion-related immune modulation [5]. It leads to a significantly higher rate of various infections, e.g., pneumonia or surgical site infections, which subsequently reduces the patients’ outcome [6,7]. Furthermore, it carries the risk of lethal transfusion incidents, as clinical errors are shown to be the most common cause of transfusion related complications [8], and it has become a decidedly scarce resource [9]. Other risks associated with ARBCT include transfusion-associated volume overload, electrolyte shifts such as hyperkalaemia and hypocalcaemia, allergic reactions, haemolysis, and transfusion-related acute lung injury (TRALI) [10]. In addition, the transmission of bacterial or viral infections due to contamination of the allogeneic blood cannot be entirely ruled out, despite significantly improved processing methods.

In 2001, the WHO developed a multidisciplinary, patient-centered concept that aims to reduce and prevent anemia and blood loss and to use blood products to a rational extent: patient blood management [1]. The three pillars of this concept include: (1) preoperative diagnosis and, if necessary, treatment of pre-existing anemia; (2) avoiding intraoperative blood loss, which includes, for example, optimizing coagulation management or autologous blood transfusion; and (3) the rational usage of ARBCT, taking into account the individual transfusion tolerance limit in the context of the patient’s constitution and underlying diseases.

Autologous blood transfusion, as included in the second pillar of patient blood management, can be accomplished using intraoperative cell salvage (ICS) devices. It represents one of the most important measures in the prevention of intraoperative blood loss [9]. In a study conducted by Roets et al., it was observed that the suppression of dendritic cell and monocyte function was significantly reduced following ICS compared to ARBCT. These findings suggest that ICS may enhance immune competence [5]. Several other studies have shown that the use of ICS is not associated with an increased risk of perioperative infections, but reduces the rate of exposure to ARBCT to a significant extent [8,9,11,12]. ICS is available as a reliable perioperative resource and bears no risk of patient mix-ups that can lead to lethal transfusion incidents. In the context of patient blood management, the use of ICS is recommended when an increased perioperative blood need is anticipated [13].

Due to the increasing age of the population and a continuously increasing rate of hip and knee arthroplasties, the number of arthroplastic revision surgeries is also constantly rising [14]. The treatment of periprosthetic joint infections (PJI) thereby poses a particular challenge. These infections are associated with multiple complications including poor patient-reported outcomes, disability, reinfection, disarticulation, and markedly increased mortality [15]. PJIs require a comprehensive therapy concept with extensive surgical rehabilitation. A two-stage exchange arthroplasty, with first resection of the infected implant, followed by 12 weeks of antibiotic therapy and reimplantation at 6-week intervals, has proven to be an efficient therapy concept [16].

The reimplantation in septic two-stage exchange arthroplasty is often a complex surgical procedure [17]. Compared to aseptic and traumatic revision arthroplasty, septic revision arthroplasty may be associated with the highest intraoperative blood loss [18].

Effective patient blood management can strongly affect the morbidity and mortality of these severely ill patients. For example, to reduce the perioperative risk of additional infections (e.g., pneumonia or surgical site reinfection) these patients could benefit from the use of ICS.

However, due to the possibility of bacterial contamination of the salvaged blood, a surgical site infection is formally a relative contraindication to the use of ICS [13,19]. Nevertheless, it has been standard clinical practice for many years to use ICS in the context of reimplantation during two-stage septic hip exchange arthroplasty, since it is assumed that the infection has been sufficiently treated [18].

To date, however, no comprehensive literature or consensus recommendations are available addressing this approach [20]. The following questions arise:

1. Is the use of ICS during reimplantation in two-stage septic exchange arthroplasty a safe procedure, which can be used to optimize patient blood management?

2. Do increased septic complications occur in the postoperative course after the use of ICS in the context of reimplantation?

Important decision-making aids for the use of ICS in the context of reimplantation in septic two-stage exchange arthroplasty to optimize patient blood management could be derived from the knowledge gained.

## 2. Results

### 2.1. Pathogens

A total of 144 patients with septic two-stage revision arthroplasties were included. In 13 (9%) out of the 144 patients, microbiological organisms were detected during reimplantation. The germ spectrum is listed in Table 1. Five (3%) of the microorganisms corresponded to the original germ of the periprosthetic infection, the remaining germs differed from the original findings.

### 2.2. Patient Characteristics, Perioperative Clinical Data, and Complications

The patients’ characteristics, perioperative clinical data, and septic complications are listed in Table 2. A total of 27 (19%) out of the 144 patients received ICS blood only; the remaining patients received at least one allogeneic blood unit.

The group of patients who received immunosuppressive medication (*p* = 0.03) had skin defects (*p* = 0.01) with the presence of other implants such as pre-existing arthroplasties or artificial heart valves (*p* = 0.03), low preoperative hemoglobin levels (*p* = 0.04), and a high volume of allogeneic blood transfusions (*p* = 0.04) demonstrated a significantly elevated rate of postoperative septic complications.

A detailed presentation of the observed septic complications during inpatient and outpatient course is shown in Table 3. Mean duration of follow-up was 46 ± 23 (0–92) months.

Two patients showed an unexplained elevation in inflammation markers after an initially falling CRP (c-reactive protein) course, which might be interpreted as a possible bloodstream-associated infection. The CRP values were in detail: for the 1st patient pre-operatively 9.4 mg/L, 5 days after surgery 110.3 mg/L, 8 days 68.0 mg/L, 19 days 221.5 mg/L, 22 days 98.4 mg/L, and falling in the following days. Temperature was a maximum of 37.3 °C during the whole period. The CRP course of the 2nd patient was pre-operatively 6.9 mg/L, 5 days after surgery 78.6 mg/L, 17 days 186.8 mg/L, 31 days 75.6 mg/L, and falling in the following days. Temperature was a maximum of 37.5 °C during the whole period. No focus of an infection was found in the extended diagnostics. The symptoms subsided in both cases without antibiotic therapy.

One patient with suspected endocarditis of the mitral valve developed an increase in the inflammation values and acute cardiac instability 14 days after reimplantation. The patient had a history of biological aortic valve replacement 14 years previously and knee arthroplasties of both knees 10 and 11 years previously, respectively. Due to an insufficiency in the mitral valve with sonographically depictable floating structures, a surgical mitral valve replacement was performed. The aortic valve and both knee arthroplasties showed no signs of peri-implant infections. The samples intraoperatively obtained during cardiac surgery did not reveal the presence of any pathogenic microorganisms or causative germ.

No patient died during the inpatient follow-up. Nine (6%) patients died during the outpatient follow-up. The mean time between reimplantation and death was 27 ± 13 (4–50) months. No death was associated with the surgery or a presumed septic complication.

Table 4 shows the frequency of septic complications depending on the intraoperative microbiological cultures. In total, no significant differences regarding the frequency of any infection and the results of the blood cultures could be determined (Chi^2^: *p* = 0.311). The Phi-Coefficient, to investigate the association between the blood culture results and any infectious complication, amounts Φ = 0.04, indicating that there is no general correlation between infection and positive blood cultures.

Considering the individual types of septic complications, no significant associations were detected in terms of non-specific septic complications (Fisher’s exact test: *p* = 0.999, Φ = 0.08), recurrent PJI/SSI (Fisher’s exact test: *p* = 0.999, Φ = 0.07), or possible bloodstream infection (Fisher’s exact test: *p* = 0.273, Φ = 0.12). Based on a further risk analysis, the relative risk (RR) for possible bloodstream infection was found to be 1.09 [95% Confidence interval 0.985 to 1.475). This result indicates that the group with positive blood cultures had a 1.09 times higher risk of developing an infection compared to the group with negative cultures during reimplantation. It is important to note that the relative risk of 1.09 suggests a modestly increased risk of infection, which, however, was not statistically significant.

## 3. Discussion

Periprosthetic infections are amongst the most serious complications of arthroplastic surgery and are associated with the high morbidity and mortality of patients [15]. Due to the heterogeneous study population with multiple influencing factors, the optimal therapy of PJI remains a challenge today and the ideal therapy concept continues to be controversially debated in the literature [21]. Surgical therapy by two-stage exchange arthroplasty, with first-stage resection arthroplasty, followed by 12 weeks of antibiotic therapy and second-stage reimplantation at 6-week intervals, has proven to be efficient in curing PJI [16]. The reimplantation arthroplasty can be a complex surgical procedure that may be associated with a long operating time and high perioperative blood loss [17].

Efficient patient blood management is considered an important measure to improve the morbidity and mortality of these severely ill patients [18]. ICS thereby represents one of the most important strategies in the prevention of intraoperative blood loss [18]. Infections of the surgical site are considered a formal contraindication to the use of ICS, as the retransfusion of potentially bacterially contaminated blood is to be avoided [13,19].

Nevertheless, the use of ICS in the context of reimplantation in two-stage septic exchange arthroplasty has been common clinical practice for many years, as it is assumed that the pre-existing infection has been treated sufficiently [18]. To date, there is no comprehensive literature and no consensus recommendation to support this approach [11].

The aim of this study was to evaluate the safety of ICS in the setting of reimplantation after successfully treated periprosthetic joint infection in septic two-stage exchange hip arthroplasty.

This double-center study was conducted at two hospitals, each of which covered the entire spectrum of primary and revision arthroplasty. It was possible to retrospectively observe a large patient collective of 144 patients.

With a mean age of more than 65 years, predominance of male sex, increased BMI, and a predominant proportion of patients with an ASA score greater than 2, the observed patient population resembles the collectives of other studies investigating PJI, indicating that these preconditions belong to the main risk factors for PJI [7,22].

There was a significantly higher rate for all kinds of septic complications in the group of patients with immunosuppression due to medication, skin defects, a low preoperative haemoglobin level, and a high number of allogeneic blood units transfused. Also this confirms the observations of previous studies that identified the named parameters as risk factors for the occurrence of septic complications in general [6,7,23]. Especially, the negative impact of preoperative anemia and of a high volume of ARBCT on the overall patients’ outcome and on all kinds of septic complications has been described before [6,7,23]. It should be noted that a preoperative low Hb value can lead to a high volume of ARBCT, which impairs an isolated evaluation of these two parameters. Given the background of multiple possible further influencing factors, no definite causal attribution of the origin of the septic complication is possible, but rather, an association of the described parameters to septic complications in general has to be described.

There was a notably higher incidence of septic complications among patients who had pre-existing implants, such as other arthroplasties or artificial heart valves. In both clinics, it was standard clinical practice to exclude PJI of other arthroplasties as part of the pre-operative diagnostic process. In case of pain and radiological abnormalities, a needle aspiration of all other arthroplasties was performed and screened for PJI. Therefore, we believe it is unlikely that the presence of an undiagnosed PJI in another implant was responsible for the septic complications observed in this study, although this cannot be ruled out with absolute certainty. The increased occurrence of septic complications in the presence of another implant might thus also be an expression of confounding influence factors, such as high patient age or many pre-existing diseases, and thus not represent a causal relationship.

In 13 (9%) out of the 144 patients, positive microbiological cultures were detected during reimplantation, with the organisms being identical to the original germ in 5 (38%) of the 13 cases. The occurrence of positive microbiological cultures during reimplantation is reported to be on average 15% [24,25,26,27,28,29,30,31,32,33,34]. Thus, the rate of positive cultures observed here is comparably low. The percentage of cultures that were identical to the original germ is reported to be on average 24% [24,25,26,27,28,29,32,33], meaning that the value observed in this study is thus comparatively high. However, due to the relatively small number of cases observed in this cohort, the significance of the observation should be interpreted with caution.

In the study presented here, all septic complications were investigated in the short-term and long-term follow-up. They were subdivided and evaluated separately in three categories: non-specific septic complications, local persistence of infection such as recurrent PJI or SSI (surgical site infection), and possible bloodstream-associated infections.

A total of 8 (6%) out of the 144 patients developed a non-specific septic complication, such as a urinary tract infection, enteritis, or pneumonia. The occurrence of these non-specific complications may be an expression of possible immunosuppression, which may be intrinsic to the patient or iatrogenic, such as due to ARBCT [6]. The occurrence of an unclear enteritis in this patient population might also be wrongly interpreted as a septic complication, since diarrhea, for example, might be an expression of a disturbed intestinal flora due to the long-lasting antibiotic treatment and thus has no infectious etiology. Taking the literature as a comparison, no increase of non-specific septic complications was observed: pneumonia and urinary tract infections were described in 2–5% of cases after primary and revision hip arthroplasty with high age, male gender, and a high number of pre-existing conditions being identified as prevalent risk factors [35,36,37].

A total of 6 (4%) out of the 144 patients showed a recurrent local infection such as persistent PJI or SSI in the follow-up. This resembles the results of previous studies investigating PJI with 3–9% of recurrence rate after two-stage exchange arthroplasty being described [38,39]. Liu et al. recently investigated the possible influence of ICS on the recurrence rate of PJI after the use of ICS at reimplantation. There was no evidence for an increased recurrence rate of PJI due to the use of ICS [39].

Of particular relevance for assessing the safety of ICS in the context of reimplantation was the occurrence of possible bloodstream-associated infections during the follow-up. In the study presented here, only in two patients could an unclear increase of the inflammation values be observed, which spontaneously subsided without antibiotic therapy. One patient developed signs of endocarditis, which was treated surgically with a mitral valve replacement. No intraoperative detection of germs during cardiac surgery was possible. This case must be considered as a possible bloodstream-associated infection, although a causal relationship cannot be established with certainty. To date, there are no comparative studies in the literature to our observations.

The presented study furthermore tested a possible association of positive intraoperative cultures with the occurrence of septic complications in the follow-up, since ICS could possibly lead to the systemic spreading of a local infection. No significant association between positive intraoperative cultures and the occurrence of any kind of septic complications during the entire follow-up after using ICS could be determined. In the literature, however, the relevance of positive microbiological cultures in the context of reimplantation remains an important topic, as a 3-fold higher risk of recurrent PJI for positive microbiological cultures compared to negative cultures has been described [24]. Interestingly, in the study presented here, none of the 6 patients with recurrent PJI or SSI had had positive intraoperative cultures at reimplantation. Ultimately, however, it always remains unclear whether positive cultures during reimplantation are an expression of contamination, persistent infection, or new PJI [40].

In summary of the results, no accumulation of septic complications due to the use of ICS during reimplantation could be observed in this study.

In the overall review of the available literature, the evidence for the use of ICS in the setting of reimplantation remains scarce. To date, only Liu et al. investigated the recurrence rate of PJI after using ICS in the context of reimplantation and could not find any increase [39]. In the present study, too, no accumulation of septic complications of any kind after the usage of ICS at reimplantation could be observed, even when positive intraoperative cultures were present. One patient showed a possible bloodstream-associated infection, although a causal attribution of the complication was not possible with certainty.

In summary of the currently available literature, including the study presented here, we consider the use of ICS during reimplantation in septic two-stage exchange arthroplasty to be indicated in terms of patient blood management and to avoid ARBCT. However, given the paucity of available literature, further studies are needed to confirm the safety of ICS in the context of reimplantation in septic revision arthroplasty.

An interesting question for future studies may be the use of ICS during active infection, for example, in the context of septic one-stage exchange arthroplasty or first-stage resection in two-stage exchange arthroplasty, as these procedures continue to be associated with high exposure to ARBCT [18]. Several studies have already shown ex vivo that the additional use of leukocyte depletion filters resulted in a strong reduction of the bacterial load in the processed ICS blood [41,42].

So far, only one clinical study mentioned the use of ICS with additional leukocyte depletion filter in the context of septic revision arthroplasty with an active infection of the surgical site [18]. It should be noted that these filters only remove organisms that have already been phagocytosed by the much larger leukocytes. Free microorganisms may be too small to be filtered out and therefore still pose a potential risk in clinical use.

However, the safety of this procedure in clinical use has not yet been investigated and might represent a possible research question for the optimization of patient blood management in the context of septic revision arthroplasty.

## 4. Materials and Methods

### 4.1. Study Design and Patients

A retrospective study was conducted at two orthopaedic centers that offer comprehensive primary and revision arthroplasty surgery, encompassing the entire range of procedures. In both hospitals, ICS was used as a standard procedure during second-stage reimplantation arthroplasty. All patients who received processed ICS blood were included in the period from July 2013 to July 2018. There were no general contraindications to the use of ICS. Only patients who did not meet the inclusion criteria were excluded; no further exclusion criteria were defined.

At both hospitals, two-stage septic exchange arthroplasty was performed according to a standardized protocol. Initially, percutaneous needle aspiration of synovial fluid with microbiological culture and determination of serological parameters, such as white blood cell count, was performed to confirm the suspected diagnosis of PJI.

First-stage resection arthroplasty included extensive debridement with collection of at least 5 solid microbiological and histological samples, removal of infected tissue, devitalized bone, and all foreign material, followed by an extensive lavage.

Empiric antibiotic therapy was administered according to a standardized protocol established in a multiprofessional collaboration with the microbiology department, depending on the local germ spectrum. If the causative microorganism was known, resistogram-based antibiotic therapy was performed. Antibiotics after first-stage implant resection and second-stage reimplantation arthroplasty were applied intravenously initially for a period of 2 weeks and then continued orally for 4 more weeks, resulting in a 12-week interval of antibiotic therapy altogether.

Second-stage reimplantation arthroplasty was performed at 6-week intervals. A second debridement with another collection of multiple microbiological and histological samples, lavage, and subsequent reimplantation arthroplasty was performed. Both hospitals used ICS as a standard procedure during reimplantation for many years, unrelated to this study. Patient data were extracted from the hospital’s internal information system. For information retrieval, the physician and consultation letters, external documentation from other hospitals, laboratory, microbiological and histological findings, as well as ward round documentations were analyzed.

Patients’ demographics, pre-existing conditions, and past medical history as well as previous medication and operation specific data, such as surgery duration, estimated blood loss, transfused ICS volume, and the units of additionally transfused allogeneic blood were recorded. The microbiological samples taken preoperatively during percutaneous needle aspiration of synovial fluid and intraoperatively during first-stage resection and second-stage reimplantation arthroplasty were examined for possible detection of microorganisms. Any positive cultures found were compared with all previous findings.

Follow-ups were assessed during the inpatient course as well as during all outpatient controls performed in the course. Possible follow-up operations and a possible death with the cause of death were documented.

All septic complications occurring during inpatient and outpatient course were documented. The septic complications were subdivided into the following three groups: non-specific septic complication, local recurrence of PJI or SSI, and possible blood stream-associated infections. A possible association between patient- or surgery-specific parameters and the detection of positive intraoperative microbiological cultures with the occurrence of septic complications was investigated.

All data were anonymously documented in an Excel file (Version 16.72, Excel for Mac, Microsoft, Redmond, WA, USA) and stored on a password-protected computer.

### 4.2. Statistical Analysis

A descriptive and inductive statistical evaluation was performed. Continuous variables are expressed as mean ± standard deviation (SD), while categorical variables are expressed as number and percentage (%). To statistically compare subgroups, appropriate statistical tests were utilized based on the nature of data. For metric data, the Kruskal–Wallis H-test was used. Categorical variables were analyzed using either the Chi^2^ or Fisher’s exact tests. To examine the association between variables, Phi-correlation analysis was employed. The Phi-correlation coefficient (Φ) is a statistical measure used to assess the strength and direction of the relationship between two categorical variables. A Phi-correlation coefficient of 0.8 would suggest a strong association, while a coefficient of 0.2 would indicate a weak association. Further analysis included the calculation of the relative risk (RR). RR is a statistical measure that quantifies the strength of association or the risk of an event occurring in one group compared to another. It is commonly used in epidemiology and clinical research to assess the impact of a particular exposure or risk factor on the likelihood of developing a specific outcome or disease. SPSS statistical program 29.0 (SPSS, Chicago, IL, USA) was used for statistical analyses. The significance level was set at 0.05 for all statistical analyses.

## 5. Conclusions

In this large collective, no increase in septic complications was observed with the use of ICS during reimplantation after PJI of the hip. One complication possibly associated to ICS was observed without a definite causal attribution.

In the risk–benefit analysis, we consider the use of ICS during reimplantation in septic two-stage exchange arthroplasty to be indicated in terms of patient blood management. Further studies are needed to further prove the safety of the procedure in the comparison of ICS to ARBCT.

The use of ICS during active infection, for example, septic one-stage exchange arthroplasty or first-stage resection in two-stage exchange arthroplasty remains a potentially interesting unanswered research question for future studies.

## 6. Limitations

Due to the low number of observed septic complications, statistical analyses of significance were only possible to a very limited extent.

Since it has been common clinical practice to use ICS in the context of reimplantation and a benefit for the patient is assumed, it was ethically not justifiable to withhold this therapy from a group of patients to create a control group. Thus, in the present study, a control group had to be omitted and a reference to the literature was drawn instead.

Due to the retrospective study design, only previously documented information could be evaluated. Furthermore, it is possible that patients continued their treatment in another hospital and thus escaped our observation.

## Figures and Tables

**Table 1:** Germ detection during reimplantation.

Germ Detection during Reimplantation	n	Identical to Original Germ
No germ detection	131	
Staph. epidermidis	5	3/5
Staph. aureus	3	1/3
Staph. capitis	3	0/3
Candida albicans	1	1/1
Candida famata	1	0/1
**total**	**144**	

**Table 2:** Patient characteristics and perioperative clinical data.

	Total n = 144	No Comp. n = 127	Non-Specific Septic Comp. n = 8	Recurrent PJI/SSI n = 6	Possible Bloodstream Inf. n = 3	*p*-Value ***
Age (yr) *	69 ± 11	69 ± 11	76 ± 6	68 ± 8	76 ± 6	0.13
Male sex **	82 (57%)	73 (58%)	3 (38%)	4 (67%)	2 (67%)	0.66
BMI (kg/m^2^) *	29 ± 6	29 ± 6	30 ± 9	30 ± 8	30 ± 4	0.98
ASA score > 2 **	82 (57%)	68 (54%)	7 (88%)	5 (83%)	2 (67%)	0.14
Diabetes **	34 (24%)	28 (22%)	3 (38%)	2 (33%)	1 (33%)	0.68
Immunosuppression due to medication **	9 (6%)	6 (5%)	1 (13%)	2 (33%)	0 (0%)	**0.03**
Skin defect/ulcer/erysipelas **	11 (8%)	7 (6%)	3 (38%)	1 (17%)	0 (0%)	**0.01**
Malignant disease **	10 (7%)	10 (8%)	0 (0%)	0 (0%)	0 (0%)	0.70
Chronic alcohol abuse **	5 (4%)	5 (4%)	0 (0%)	0 (0%)	0 (0%)	0.88
Presence of other implants (arthroplasty, artificial heart valve) **	44 (31%)	36 (28%)	4 (50%)	1 (17%)	3 (100%)	**0.03**
Preop. haemoglobin (g/dL) *	11.4 ± 1.7	11.6 ± 1.8	10.4 ± 1.2	11.1 ± 0.9	9.9 ± 1.6	**0.04**
Surgery duration (min) *	125 ± 45	124 ± 45	123 ± 48	125 ± 29	161 ± 86	0.74
Estimated blood loss (mL) *	1910 ± 906	1948 ± 880	1256 ± 708	2455 ± 1323	2110 ± 156	0.08
Volume of transfused ICS blood (mL) *	462 ± 264	473 ± 270	292 ± 139	412 ± 229	523 ± 273	0.43
Total number of allogeneic blood units transfused *	2.6 ± 2.1	2.4 ± 1.9	4.1 ± 3.0	3.3 ± 3.8	5.7 ± 1.5	**0.04**

* Presentation of the values as mean and standard deviation. ** Presentation of the values as total amount and percentage. *** *p*-values assessed by Person Chi-square or Kruskal–Wallis test, as appropriate.

**Table 3:** Septic complications during inpatient and outpatient course.

**No Septic Complications**	**127**
**Non-specific septic complication**	**8**
- Urinary tract infection	5
- Enteritis	2
- Pneumonia	1
**Recurrent PJI/SSI**	**6**
- Recurrent PJI	4
- Wound abscess	1
- Phlegmon of the quadriceps femoris muscle	1
**Possible bloodstream-associated infection**	**3**
- unclear increase in inflammation values with no apparent focus	2
- suspected endocarditis of the mitral valve	1
**total**	**144**

**Table 4:** Association of septic complications with intraoperative microbiological cultures.

	No Comp.	Non-Specific Septic Comp.	Recurrent PJI/SSI	Possible Bloodstream Inf.	Total
Negative cultures during reimplantation	115	8	6	2	**131**
Positive cultures during reimplantation	12	0	0	1	**13**
**total**	**127**	**8**	**6**	**3**	**144**

## Data Availability

Data are available from the authors on reasonable request.
